# Efficacy and safety evaluation of dexmedetomidine for postoperative patient controlled intravenous analgesia: A systematic review and meta-analysis

**DOI:** 10.3389/fphar.2022.1028704

**Published:** 2022-12-12

**Authors:** Rui Chen, Shujun Sun, Yufan Li, Xiaoke Dou, Maosha Dai, Yan Wu, Yun Lin

**Affiliations:** ^1^ Department of Anesthesiology, Union Hospital, Tongji Medical College, Huazhong University of Science and Technology, Wuhan, China; ^2^ Department of Rehabilitation Medicine, The People’s Hospital of Honghu, Honghu, China; ^3^ Department of Dermatology, Union Hospital, Tongji Medical College, Huazhong University of Science and Technology, Wuhan, China

**Keywords:** dexmedetomidine, patient controlled intravenous analgesia, postoperative analgesia, randomized controlled trail, meta-analysis

## Abstract

**Objective:** To investigate the efficacy and safety of dexmedetomidine (DEX) for postoperative patient controlled intravenous analgesia (PCIA).

**Measurements:** Two investigators independently searched Pubmed, Embase, Scopus, Cochrane Library and CBM for randomized controlled trials of DEX for PCIA.

**Main results:** Thirty-seven studies with a total of 5,409 patients were included in this meta-analysis. Compared with analgesics alone, DEX for PCIA reduced pain score at 24 h [mean difference (MD) = −0.70; 95% confidence interval (CI): −0.85, −0.54; *p* < 0.00001, *I* ^*2*^ = 90%] and 48 h postoperatively (MD = −0.43; 95% CI: −0.52, −0.34; *p* < 0.00001, *I* ^*2*^ = 96%). Moreover, DEX reduced analgesics consumption during the first 24 h [standardized mean difference (SMD) = −0.25; 95% CI: −0.34, −0.16; *p* < 0.00001, *I* ^*2*^ = 91%] and the number of resuscitation analgesics administered [odds ratio (OR) = 0.54; 95% CI: 0.44, 0.66; *p* < 0.00001, *I* ^*2*^ = 72%]. Furthermore, DEX improved patient satisfaction (OR = 3.55; 95% CI: 2.36, 5.35; *p* < 0.00001, *I* ^*2*^ = 60%), and reduced incidence of side effects, such as postoperative nausea and vomiting (PONV) (OR = 0.47; 95% CI: 0.39, 0.57; *p* < 0.00001, *I* ^*2*^ = 59%) and pruritus after surgery (OR = 0.45; 95% CI: 0.30, 0.68; *p* = 0.0001, *I* ^*2*^ = 0%). Besides, DEX attenuates inflammatory cytokine levels, such as IL-6 (MD = −5.73; 95% CI: −8.34, −3.12; *p* < 0.00001, *I* ^*2*^ = 91%) and TNF-α (MD = −0.63; 95% CI: −0.76, −0.50; *p* < 0.00001, *I* ^*2*^ = 89%). Finally, DEX increased the risk of bradycardia (OR = 1.66; 95% CI: 1.12, 2.45; *p* = 0.01, *I* ^*2*^ = 15%), but the complication of hypotension did not differ between the two groups (OR = 1.30; 95% CI: 0.84, 2.04; *p* = 0.25, *I* ^*2*^ = 0%).

**Conclusion:** DEX is used for postoperative PCIA analgesia, which can significantly improve the analgesic effect, effectively control postoperative inflammatory response, reduce the dosage and adverse reactions of analgesics, and improve postoperative patient satisfaction. Of course, the impact of the immunosuppressive effect of DEX on the prognosis of patients needs further study.

**Systematic review registration:** CRD42022340933, https://www.crd.york.ac.uk/prospero/.

## Introduction

Patient controlled analgesia (PCA) is currently the most commonly used and ideal method for postoperative analgesia, with rapid onset of action, no analgesic blind spots, relatively stable blood concentrations, and timely control of burst pain by pulse (bolus) dose, meanwhile, it has the advantages of individualized medication and high patient satisfaction, and is suitable for moderate to severe pain after surgery (Pamela and Ronaldo, 2010) Patient controlled intravenous analgesia (PCIA) means that when the patient feels pain, press the start button (bolus) in the PCA pump to inject a set dose of drugs intravenously into the body through a computer-controlled micropump, which commonly used analgesics include opioids (morphine, oxycodone, hydromorphone, sufentanil, hydrocodone, fentanyl, butorfenol, desocine, etc.), tramadol or flurbiprofen axetil, ketorolac, etc. (Jeffrey, 2005). However, the single-drug application of PCIA often limits their widespread use due to poor pain control or the obvious adverse reactions associated with increasing the dose of analgesic drugs, for example, increasing the dosage of opioids in order to improve the analgesic effect often causes adverse reactions such as itching, vomiting, nausea and even respiratory depression (Motamed, 2022). Therefore, PCIA is based on analgesic drugs and is compatible with another adjuvant drug, which can improve the analgesic effect and reduce adverse drug reactions through synergistic effects, has become a popular PCA analgesic program worldwide (Song et al., 2013).

Dexmedetomidine (DEX) is a new type of highly selective α_2_ adrenergic receptor agonist with sedative, analgesic, anxiolytic and sympathetic inhibitory effects, and no obvious respiratory depression. Multiple meta-analyses have shown that DEX application can significantly reduce the amount of sedative and analgesic drugs, reduce the occurrence of adverse reactions of general anesthesia drugs, and provide better sedative and analgesic effects (Tsaousi et al., 2018; Wang et al., 2018). However, most of the above meta-analyses focus on the application of DEX in general anesthesia, its duration of action is relatively short, and the efficacy and safety of long-term use of DEX in PCIA is still confusing, worthy of attention.

Currently, there are limited studies evaluating the advantages or disadvantages of DEX in PCIA from multicenter large randomized controlled trials. Therefore, it is necessary to conduct a meta-analysis to evaluate the safety and efficacy of DEX in PCIA to provide guidance for clinical anesthesia practice.

## Methods

The protocol of this meta-analysis was registered in the International Prospective Register of Systematic Reviews (PROSPERO) with the registration number CRD42022340933. The date of registration was 16 July 2022.

### Searching strategy

Two researchers searched PUBMED, EMBASE, Scopus, Cochrane library and CBM databases independently. The mesh and keywords used for the searches included: “dexmedetomidine,” “patient controlled intravenous analgesia,” and “randomized controlled trial,” and we used an advanced search and limited it to Title/Abstract. The latest search was completed on 17 November 2022, and see the annex for the detailed search strategy. Furthermore, the investigators scanned references of these articles to prevent missing articles.

### Study selection

Original studies included were based on PICOS (patient, intervention, comparison, outcome, and study design) as follows, P: postoperative patient received PCIA; I: the use of DEX for PCIA; C: DEX versus controls; O: pain score related indicators (visual analogue scale, VAS; numerical rating scale, NRS; verbal rating scale, VRS; Wong-Baker faces pain scale revision, FPS-R), sedation score, side effects, and so on; S: only randomized controlled trials (RCTs) were included. Studies with following characteristics were excluded: 1) use of DEX for PCIA in non-surgical patients; 2) incomplete and duplicate publications; 3) data cannot be retrieved or converted; 4) non English or Chinese articles.

### Data extraction

The two reviewers screened the literature independently. First, duplicate articles were deleted through Endnote (version X7.7.1) and manual methods. Then, articles that met the inclusion criteria were selected by reading the title, abstract, or even full text. Further, relevant data were extracted from the included articles according to the pre designed data extraction table, and cross checked. The pre designed data table includes the name of the first author, the year of publication, the sample size of each group of patients, the interventions of experimental and control group, and the type of surgery. And the following data from the included studies will be extracted for further statistical analysis: 1) pain score at different time points after operation; 2) analgesics consumption during the first 24 postoperative hours; 3) patient satisfaction; 4) the adverse reactions (nausea, vomiting, pruritus, hypotension and bradycardia); 5) sedation scores at different time points postoperatively; 6) changes in inflammatory signals (IL-6 and TNF-α).

### Assessment of risk of bias

Two reviewers independently read and evaluated the methodological validity of all eligible studies using the Cochrane Handbook v5.0.2. When disagreements arose, they were resolved through joint consultations. If necessary, a third researcher assisted in the decision. The following information was evaluated: random sequence generation, allocation concealment, blinding, incomplete outcome data, selective reporting, and other biases. They were rated as “high risk of bias,” “uncertain risk of bias,” and “low risk of bias,” respectively.

### Statistical analysis

All included RCTs were quantitatively analyzed using Review Manager (version 5.3; Cochrane Collaboration, Copenhagen, Denmark) software, and Stata 12.0 (StataCorp, College Station, TX, United States) was used for Begg’s test. Binary outcomes were computed as odds ratios (OR) with 95% confidence interval (95% CI), and continuous outcomes were calculated as the mean difference (MD) or the standardized mean difference (SMD) with 95% CI. In terms of heterogeneity analysis: a fixed-effects model was used to analyze non-significant heterogeneity data (*I* ^*2*^ < 50). Otherwise, the random effects model is used for calculation. If the heterogeneity is significant, look for possible influencing factors of heterogeneity, and try to exclude the influence of heterogeneity through subgroup analysis, sensitivity analysis and other methods.

## Results

### Characteristics and risk of bias of eligible trials

**FIGURE 1:**
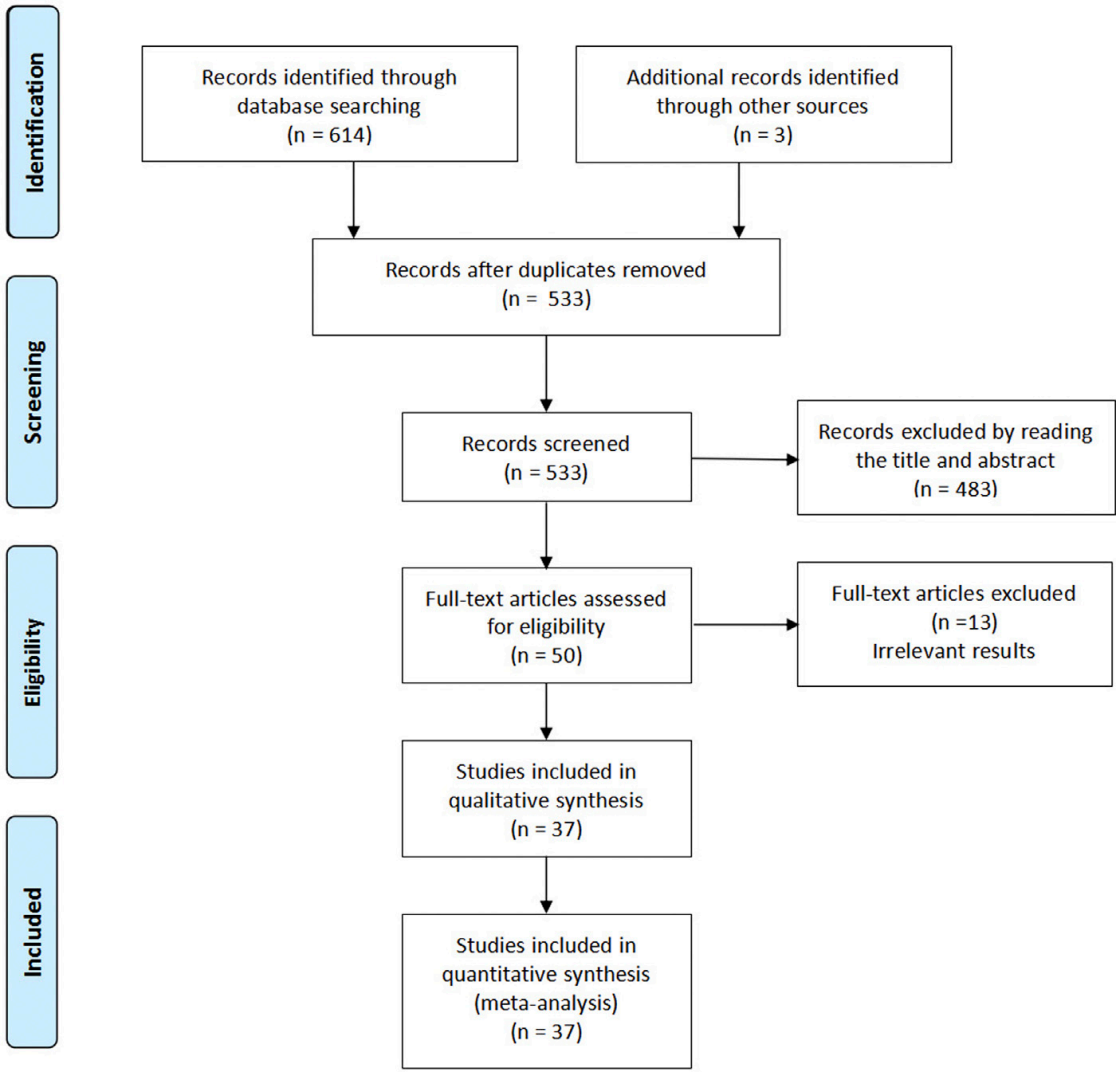
Flow chart for article selection in the meta-analysis.

**FIGURE 2:**
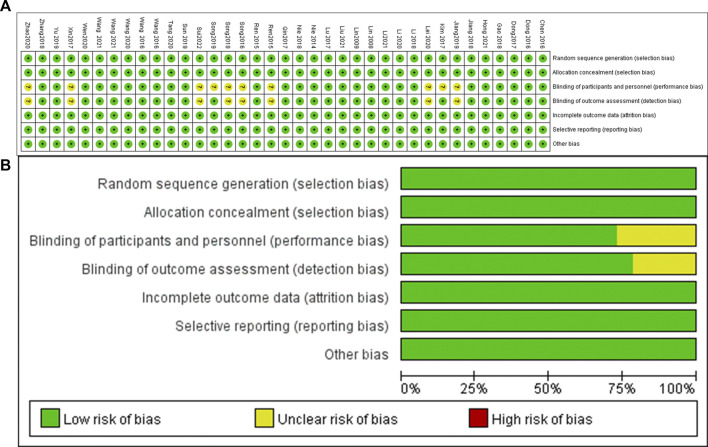
The risk of bias assessment of the included studies. **(A)** Risk bias of summary, **(B)** Risk bias of graph. Note: There were no high risk of bias found in these studies.

Our research flow chart is shown in Figure 1. A total of 37 RCTs were identified, including 5,409 patients. The risk of bias assessment was fully consistent between the two reviewers and showed moderate overall study quality. As shown in Figure 2, the risk of bias map was created using ReviewManager 5.3 software.

### Study characteristics

The study characteristics are presented in Table 1. The included studies were first published in 2008 and the sample sizes ranged from 12 to 351 patients. In the 37 studies included in this meta-analysis, PCIA were used in abdominal surgeries (seven studies), cesarean surgeries (five studies), laparoscopic surgery (two studies), spine surgeries (two studies), shoulder arthroscopy (one study), and other procedures (20 study).

**TABLE 1:** Characteristics of included studies.

Author (year)	Sample size		Intervention study		Surgery type
	Experimental group	Control group	Experimental group	Control group	
Lin et al. (2009)	50	48	Morphine 1 mg/ml + DEX 5 μg/ml	Morphine 1 mg/ml	Hysterectomy
Lin et al. (2009)	50	50	Morphine 1 mg/ml + DEX 5 μg/ml	Morphine 1 mg/ml	Abdominal surgery
Nie et al. (2014)	38	38	Sufentanil 100μg + DEX 300 μg	Sufentanil 100 μg	Cesarean Surgery
Ren et al. (2015)	60	30	Sufentanil 0.02 mg/kg/h + DEX 0.02, 0.05 μg/kg/h	Sufentanil 0.02 mg/kg/h	Abdominal Surgery
Ren et al. (2015)	84	41	Sufentanil 0.02 mg/kg/h + DEX 0.02, 0.04 μg/kg/h	Sufentanil 0.02 mg/kg/h	Thoracic Surgery
Dong et al. (2016)	12	13	Sufentanil 3 μg/kg + DEX 3.0 μg/kg	Sufentanil 3 μg/kg	Spine Surgery
Chen et al. (2017)	30	29	Sufentanil 0.02 μg/kg + DEX 0.05 μg/kg	Sufentanil 0.02 μg/kg	Abdominal Surgery
Song et al. (2019)	53	52	Fentanyl 10 μg/kg + 120 mg Ketorolac + DEX 10 μg/kg	Fentanyl 10 μg/kg + 120 mg Ketorolac	--------------------------
Wang et al. (2016a)	20	16	DEX 0.25 μg/kg/h	Fentanyl 20 mg/kg	Gynecological Surgery
Wang et al. (2016b)	42	42	Oxycodone 50 mg + DEX 0.05 μg/kg/h	Oxycodone 50 mg	Thoracoscopic Lobectomy
Dong et al. (2017)	30	30	Sufentanil 3.0 μg/kg + DEX 4.0 μg/kg	Sufentanil 3.0 μg/kg	Thoracotomy Surgery
Kim et al. (2017)	57	57	Fentanyl 0.03 µg/kg + DEX 0.007 µg/kg	Fentanyl 0.03 µg/kg	Gastrectomy
Lu et al. (2017)	76	76	Sufentanil 0.04 μg/kg/h + DEX 0.06 μg/kg/h	Sufentanil 0.04 μg/kg/h	Shoulder Arthroscopy
Qin et al. (2017)	29	29	Sufentanil 1.0 μg/ml + DEX 4 μg/ml	Sufentanil 1.0 μg/ml	Partial laryngectomy
Xin et al. (2017)	46	47	Sufentanil 0.02 ug/(kg·h) + DEX 0.04 ug/(kg·h)	Sufentanil 0.04 ug/(kg·h)	Laparotomy surgery
Gao et al. (2018)	102	101	Sufentanil 100μg + DEX 200 μg	Sufentanil 100 μg	Abdominal Surgery
Jiang et al. (2018)	66	33	Oxycodone 0.6 mg/kg + DEX 2.4, 4.8 μg/kg	Oxycodone 0.6 mg/kg	Abdominal Surgery
Li et al. (2018)	28	29	Morphine 0.5 mg/ml + DEX 2 μg/ml	Morphine 0.5 mg/ml	Abdominal Surgery
Nie et al. (2018)	103	102	Sufentanil 100μg + DEX 300 μg	Sufentanil 100 μg	Cesarean Surgery
Song et al. (2019)	60	30	Sufentanil 0.02 μg/kg/h + DEX 0.02, 0.04 μg/kg/h	Sufentanil 0.02 μg/kg/h	Laparoscopic nephrectomy
Zhang et al. (2018)	26	26	Oxycodone 60 mg + DEX 360 µg	Oxycodone 60 mg	Open Hepatectomy
Jiang et al. (2018)	30	30	Sufentanil 100µg + 200 µg DEX	Sufentanil 100 µg	Burn surgery
Sun et al. (2019)	281	276	Sufentanil 2 μg/kg + DEX 4.8 μg/kg	Sufentanil 2 μg/kg	Elderly patients with non-cardiac surgery
Song et al. (2019)	30	30	Oxycodone 50 mg + DEX 2.5 μg/kg	Oxycodone 50 mg	Abdominal Surgery
Yu et al. (2019)	276	281	Sufentanil 150μg + DEX 150 μg	Sufentanil 150 μg	Cesarean Surgery
Lei et al. (2020)	57	61	Morphine 0.48 mg/kg + DEX 1.0 μg/kg	Morphine 0.48 mg/kg	Post-thoracotomy surgery
Li et al. (2020)	61	61	Morphine 0.5 mg/ml + DEX 1 μg/ml	Morphine 0.5 mg/ml	Gynecological Laparoscopic Surgery
Tang et al. (2020)	27	26	Sufentanil 1 μg/ml + DEX 2.5 μg/ml	Sufentanil 1 μg/ml	Esophageal Surgery
Wang et al. (2020)	72	73	Sufentanil 1.5 μg/kg + DEX 2 μg/kg	Sufentanil 1.5 μg/kg	Cesarean Surgery
Miao et al. (2020)	26	28	Ketorolac 3 mg/kg + DEX 0.1 µg/kg/h	Ketorolac 3 mg/kg + Sufentanil 1.5 μg/kg	Lung Cancer Surgery
Zhao et al. (2020)	315	101	Sufentanil 150µg + DEX 100, 200, 400 µg	Sufentanil 150 µg	-------------------------
Hong et al. (2021)	351	347	Sufentanil 200μg + DEX 200 μg	Sufentanil 200 μg	Orthopedic Surgery
Liu et al. (2021)	86	28	Butorphanol 3 μg/kg/h + DEX 0.03, 0.05, 0.08 μg/kg/h	Butorphanol 3 μg/kg/h	Cesarean Surgery
Li et al. (2021)	72	71	Sufentanil 1.5 μg/kg + Dezocine 0.3 mg/kg + DEX 3.0 μg/kg	Sufentanil 1.5 μg/kg + Dezocine 0.3 mg/kg	Thoracoscopic Surgery
Wang J. et al. (2021)	36	36	Fentanyl 20 µg/kg + DEX 2.5 µg/kg	Fentanyl 20 µg/kg	------------------------
Wang Y. et al. (2021)	24	25	Sufentail 0.03 μg/kg + DEX 0.03 μg/kg	Sufentail 0.03 μg/kg	Spine Surgery
Sui et al. (2022)	140	70	Sufentanil 150 μg + DEX 200, 400 μg	Sufentanil 150 μg	Colorectal cancer surgery

Abbreviations: DEX, Dexmedetomidine.

### Pain score for postoperative patients at different time points

**FIGURE 3:**
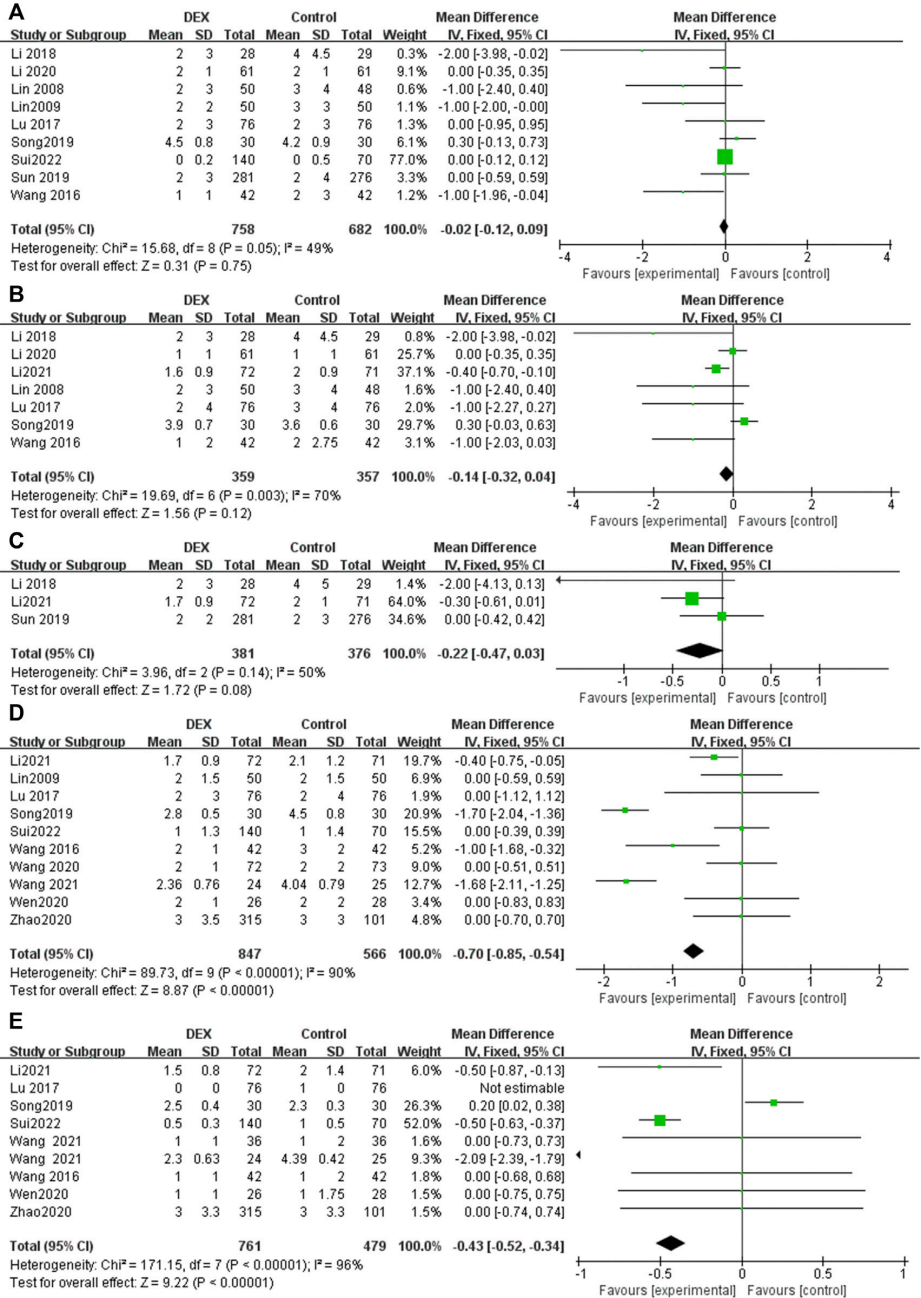
Forest plot for pain score in postoperative PCIA patients at different time points. **(A)** Pain score at 0–4 h, **(B)** Pain score at 4–8 h, **(C)** Pain score at 12 h, **(D)** Pain score at 24 h, **(E)** Pain score at 48 h.

At least seven trials reported pain score for postoperative patients at different time points (0–4 h, 4–8 h, 24 h, 48 h), while three trials reported 12 h pain score after surgery (Figure 3). Significant heterogeneity was observed among the studies in the pooled analysis at 4–8 h (*p* = 0.003, *I* ^*2*^ = 70%), 24 h (*p* < 0.00001, *I* ^*2*^ = 90%) and 48 h (*p* < 0.00001, *I* ^*2*^ = 96%). There was no statistical difference in the results at 0–4 h (MD = −0.02; 95% CI: −0.12, 0.09; *p* = 0.75), 4–8 h (MD = −0.14; 95% CI: −0.32, 0.04; *p* = 0.12) and 12 h (MD = −0.22; 95% CI: −0.47, 0.03; *p* = 0.08), however, the pain score of the patients in the DEX group was significantly lower than that in the control group postoperative 24 h (MD = −0.70; 95% CI: −0.85, −0.54; *p* < 0.00001) and postoperative 48 h (MD = −0.43; 95% CI: −0.52, −0.34; *p* < 0.00001). The above results show that, compared with a single analgesics, DEX for PCIA can significantly prolong the postoperative analgesia time and reduce postoperative pain in patients.

### Ramsay score at postoperative 24 h

**FIGURE 4:**
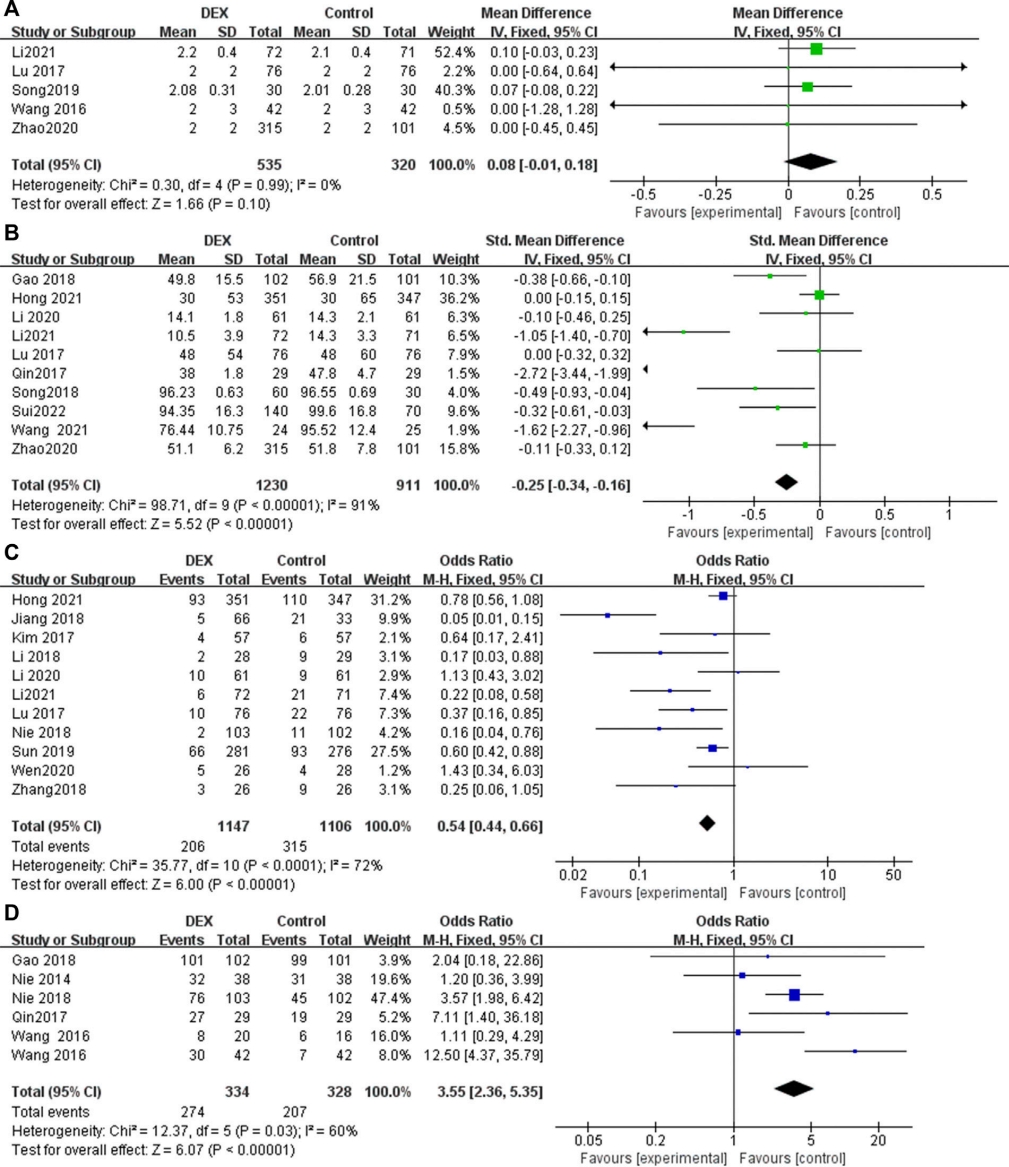
Forest plot for sedation, analgesic drug consumption, rescue analgesia and patient satisfaction in postoperative PCIA patients. **(A)** Ramsay score at 24 h postoperatively, **(B)** Total consumption of analgesics within 24 h, **(C)** Use of rescue analgesics, **(D)** Patient satisfaction.

Five RCTs reported the ramsay score at 24 h postoperatively, with low heterogeneity among the results (*I* ^*2*^ = 0%), and the fixed effects model was used for meta-analysis (Figure 4). The results showed that there was no statistical difference in the ramsay score between the two groups (MD = 0.08; 95% CI −0.01, 0.18; *p* = 0.10, Figure 4A).

### Total consumption of analgesics within 24 h

Ten studies reported supplemental analgesics consumption during the first 24 h postoperatively. The pooled results indicated that patients receiving DEX for postoperative PCIA exhibited a significant reduction in total supplemental analgesics consumption at 24 h postoperatively, compared with patients receiving analgesics alone (SMD = −0.25; 95% CI: −0.34, −0.16; *p* < 0.00001, *I* ^*2*^ = 91%, Figure 4B).

### Use of rescue analgesics

Eleven studies reported the number of times rescue analgesics were used. The pooled results indicated that patients receiving DEX for postoperative PCIA exhibited a significant reduction in use of rescue analgesics, compared with patients receiving analgesics alone (OR = 0.54; 95% CI: 0.44, 0.66; *p* < 0.00001, *I* ^*2*^ = 72%, Figure 4C).

### Patient satisfaction

Six studies reported patient satisfaction with PCIA use. Postoperative PCIA patients using DEX were significantly more satisfied than patients using analgesics alone (OR = 3.55; 95% CI: 2.36, 5.35; *p* < 0.00001, *I* ^*2*^ = 60%, Figure 4D).

### IL-6 at postoperative 24 h

**FIGURE 5:**
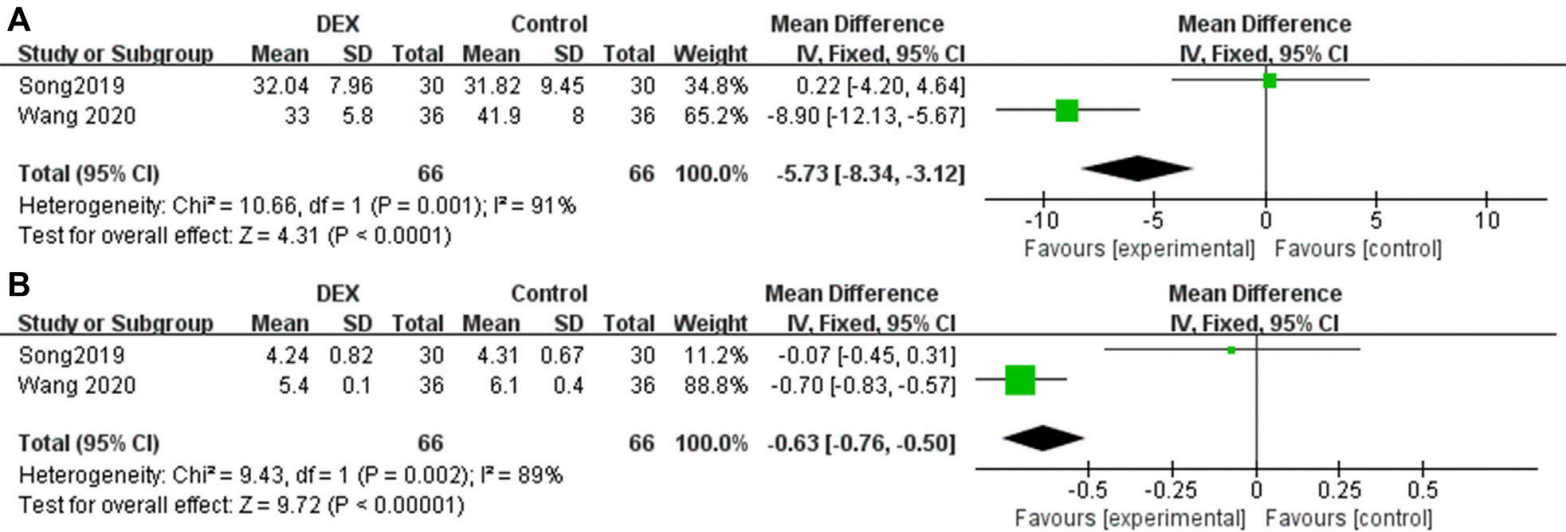
Forest plot for inflammatory levels in postoperative PCIA patients. **(A)** IL-6 at 24 h postoperatively, **(B)** TNF-α at 24 h postoperatively.

Two studies reported IL-6 at postoperative 24 h. Compared with patients who received PCIA alone with analgesics or placebo, patients who added DEX significantly decreased the inflammatory cytokine IL-6 at 24 h after surgery (MD = −5.73; 95% CI: −8.34, −3.12; *p* < 0.00001, *I* ^*2*^ = 91%, Figure 5A).

### TNF-α at postoperative 24 h

Two studies reported TNF-α at postoperative 24 h. Compared with patients who received PCIA alone with analgesics or placebo, patients who added DEX significantly decreased the inflammatory cytokine TNF-α at 24 h after surgery (MD = −0.63; 95% CI: −0.76, −0.50; *p* < 0.00001, *I* ^*2*^ = 89%, Figure 5B).

### Postoperative nausea and vomiting

**FIGURE 6:**
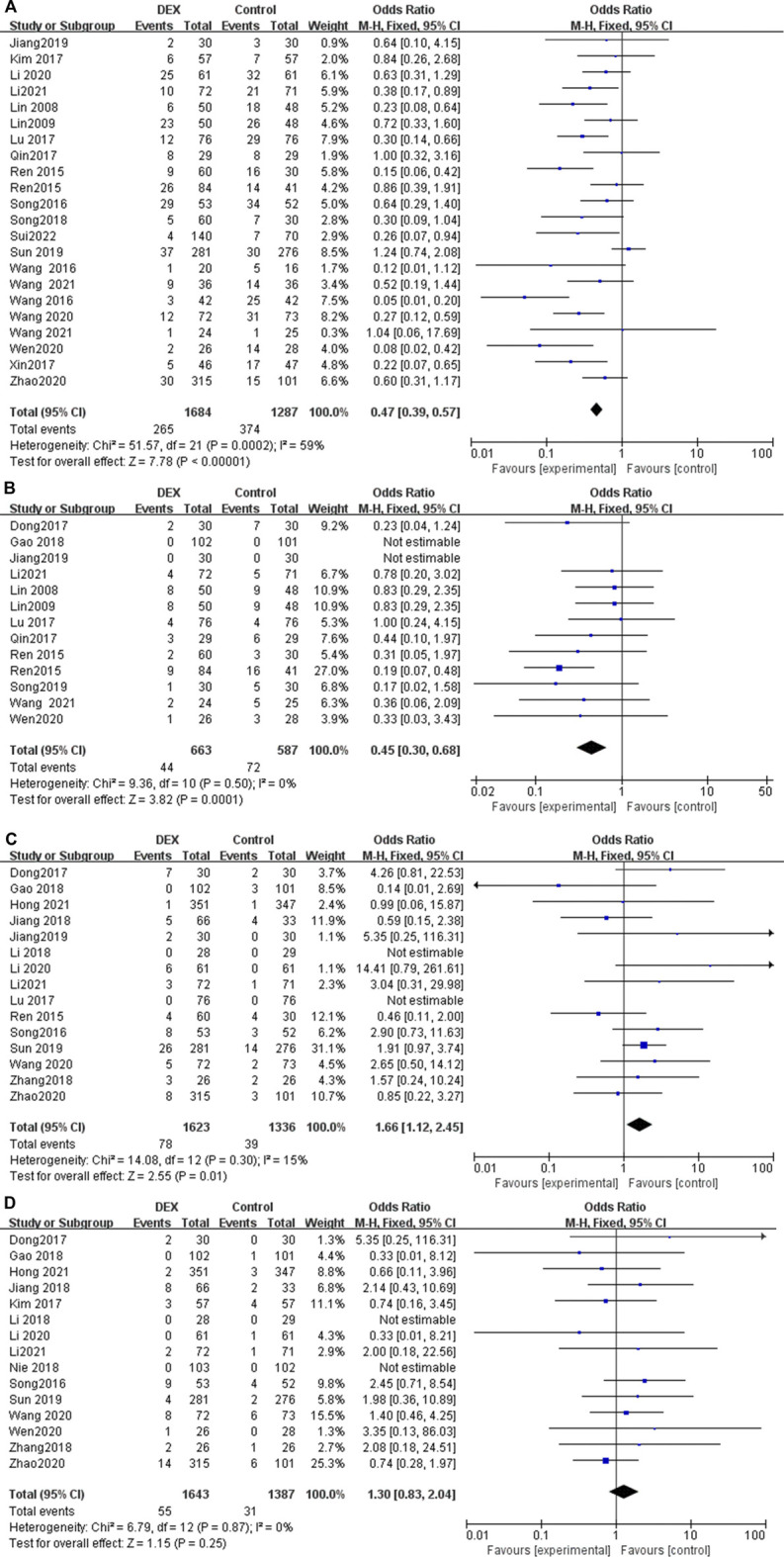
**(C)** Bradycardia after surgery, **(D)** Hypotension after surgery.

Twenty-two studies reported the incidence of nausea and vomiting in patients undergoing PCIA. The results show that the use of DEX for PCIA can significantly reduce the incidence of postoperative nausea and vomiting, compared with PCIA alone with analgesics (OR = 0.47; 95% CI: 0.39, 0.57; *p* < 0.00001, *I* ^*2*^ = 59%, Figure 6A).

### Pruritus after surgery

Thirteen trials reported the incidence of pruritus after using PCIA. Heterogeneity among the studies was insignificant in the pooled analysis (*I* ^*2*^ = 0%). Our meta-analysis found that the incidence of pruritus was significantly lower in the PCIA group with the addition of DEX compared to PCIA with analgesics alone (OR = 0.45; 95% Cl: 0.30, 0.68; *p* = 0.0001, Figure 6B).

### Bradycardia after surgery

Fifteen RCTs reported the incidence of bradycardia after using PCIA, with low heterogeneity among the results (*I* ^*2*^ = 15%). The incidence of bradycardia in the DEX group was slightly higher than that in the control group (OR = 1.66; 95% CI: 1.12, 2.45; *p* = 0.01, Figure 6C).

### Hypotension after surgery

Fifteen RCTs reported the incidence of hypotension after using PCIA, with low heterogeneity among the results (*I* ^*2*^ = 0%). The results showed that there was no statistical difference in the incidence of hypotension after using PCIA between the two groups (OR = 1.30; 95% CI 0.83, 2.04; *p* = 0.25, Figure 6D).

### Sensitivity analysis and subgroup analysis

Sensitivity analysis and subgroup analysis were used to find sources of heterogeneity, and minimize the impact of heterogeneity on the stability of results. The heterogeneity of some indicators (4–8 h pain score, PONV, use of rescue analgesics) were significantly reduced among other studies after removal of one studies, while the total consumption of analytics, 24 h and 48 h pain score reduce heterogeneity to some extent through subgroup analysis, as shown in Table 2. For indicators that total consumption of analgesics within 24 h after surgery, PONV, 24 h and 48 h pain score, the result of sensitivity or subgroup analysis were consistent with previous results. By sensitivity analysis, there was a statistically significant difference between the two groups for 4–8 h pain score, showing that the DEX group was significantly lower than the control group, contrary to previous results.

**TABLE 2:** The results of sensitivity or subgroup analysis.

Indicators	MD/OR	P	I (%)
Sensitivity analysis			
Pain score at 4–8 h postoperatively	−0.33 [−0.54, −0.12]	0.003	49
Postoperative nausea and vomiting	0.40 [0.33, 0.49]	<0.0001	46
Use of rescue analgesics	0.59 [0.48, 0.73]	<0.0001	48
Subgroup analysis			
Total consumption of analgesics			
Abdominal surgery	−0.32 [−0.48, −0.15]	0.0001	0
Non-abdominal surgery	−0.26 [−0.38, −0.14]	<0.0001	96
Pain score at 24 h postoperatively			
Thoracic surgery	−0.46 [−0.75, −0.17]	0.002	46
Non-thoracic surgery	−0.79 [−0.97, −0.61]	<0.0001	93
Pain score at 48 h postoperatively			
Published in 2020 and before 2020	0.17 [0.00, 0.33]	0.04	0
Published after 2020	−0.70 [−0.81, −0.59]	<0.00001	97

Abbreviations: MD, mean difference; OR, odds ratio.

### Publication bias

**FIGURE 7:**
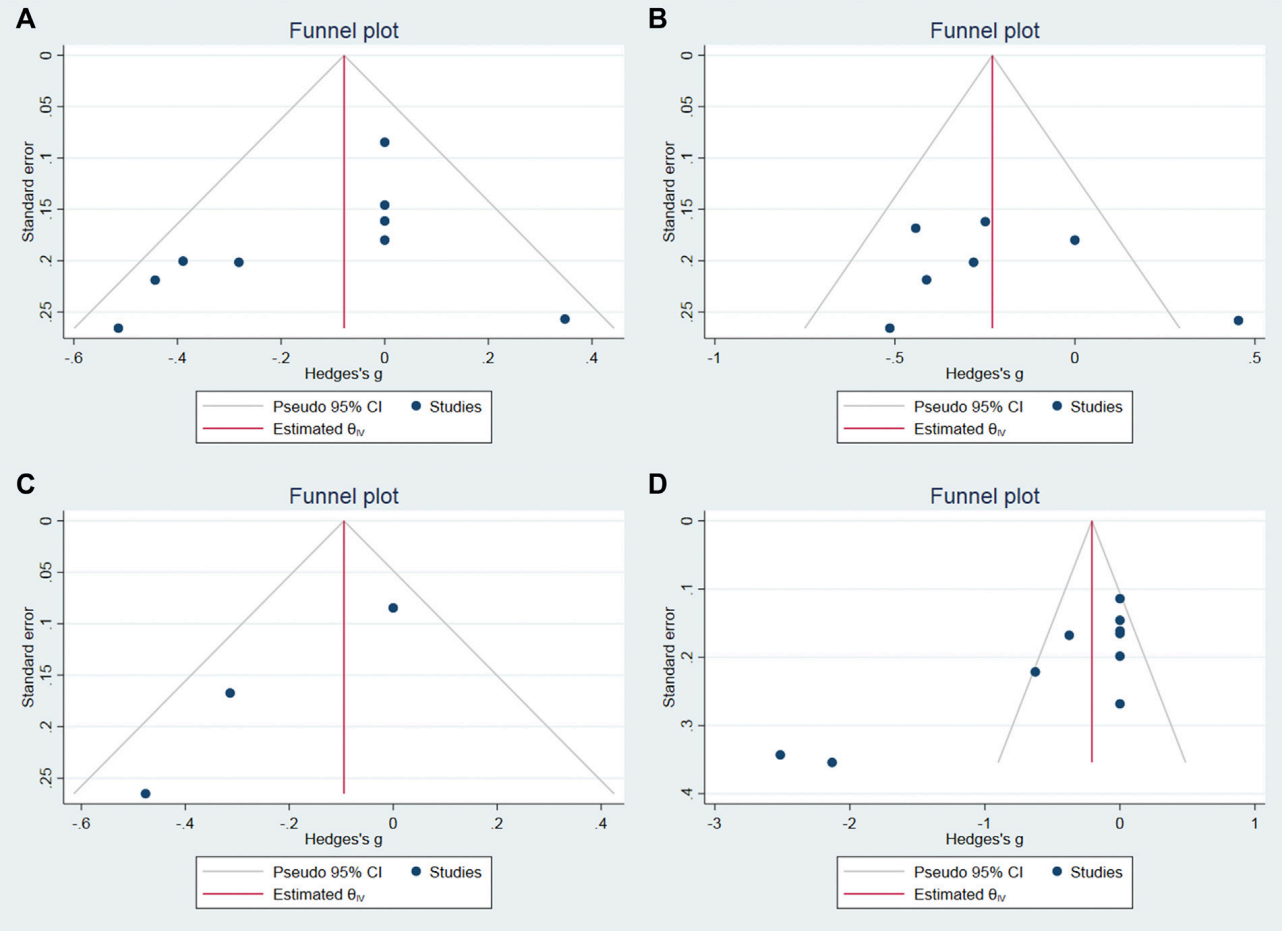
Funnel plot for pain score at 0–4 h **(A)**, 4–8 h **(B)**, 12 h **(C)** and 24 h **(D)**.

We used Stata software to assess the publication bias of the main results. The *p* values of begg’s test on the 0–4 h, 4–8 h, 12 h, and 24 h pain score were 0.0763, 1, 0.2963, 0.0013, respectively. Therefore, we believe that the risk of publication bias is low in this meta-analysis, and the funnel plot is shown in Figure 7.

## Discussion

This meta-analysis of quantitative studies of DEX for postoperative PCIA shows that DEX improved the analgesic effect, reduces total analgesics consumption, and reduced the analgesic drug-related adverse reactions. Compared with the control group, the pain score in the DEX group were significantly reduced at 24 and 48 h after surgery. In addition, DEX can also significantly improve patient satisfaction and reduce the time of analgesic rescue needs. Also, the analgesics related side effects in the DEX group, such as nausea, vomiting and itching after surgery, were significantly reduced. Moreover, DEX could reduce the levels of inflammatory cytokines such as IL-6 and TNF-α. Ultimately, DEX increased the risk of bradycardia, with no difference in hypotensive complications between the two groups. Based on the results of this meta-analysis, we summarized the specific role of DEX in PCIA, as shown in Table 3.

**TABLE 3:** The summary of the efficacy of DEX in PCIA.

Dexmedetomidine versus control in PCIA							
Effectiveness			Adverse reactions			Inflammatory levels	
Analgesic effects	Sedative effects	Patient satisfaction	Nausea vomiting Pruritus	Bradycardia	Hypotension	IL-6	TNF-α
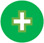	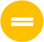	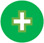	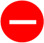	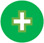	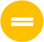	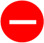	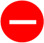

Note: 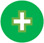
means that DEX increased the effect vs. control group; 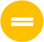
means that DEX has the same effect vs. control group; 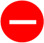
means DEX decreased the effect vs. control group. Abbreviations: PCIA, patient controlled intravenous analgesi.

The significant pharmacological effects of DEX are due to the activation of α_2_-ARs. DEX can activate presynaptic α_2_-ARs, inhibit the release of norepinephrine through a negative feedback mechanism, and stop pain signaling (Feng et al., 2019; Liu et al., 2021). The unique “conscious calming” effect of DEX is primarily related to the nucleus locus coeruleus in the brain. Compared with analgesics, it has superior properties, especially in arousal sedation, mild analgesia, and a lower risk of respiratory depression (Hall et al., 2000). In this study, DEX, as an adjuvant for analgesics, could enhance postoperative analgesia and improve patient satisfaction.

PCA is one of the accepted methods for the management of postoperative pain. A cochrane review concluded that PCA was associated with better postoperative pain scores and increased patient satisfaction compared with traditional modes of analgesic drug administration (Abrolat et al., 2018). Intravenous PCA has been widely used for postoperative analgesia over the past few decades (Hadi et al., 2006; Kim et al., 2017).

Pain can be assessed by self-assessment scales, behavioural tests and physiological measures. One-dimensional pain scales, which are quick to assess, concise and easy for patients to understand, are the most commonly used types of pain assessment scales in clinical practice and include the VAS, NRS, VRS, and FPS-R. Of the 37 studies included in our meta-analysis, Sun et al. (2019) and Li et al. (2018) used the NRS, and Lin et al. (2009) used the VRS to assess patients’ postoperative pain, while the remaining studies all used the VAS to assess pain. This meta-analysis found that the addition of DEX resulted in lower pain scores than analgesia alone at 24 and 48 h postoperatively. An interesting phenomenon found in this study is that DEX is not significantly better than analgesics in reducing the pain score of patients 0–12 h after surgery. Intravenous analgesics given prior to switching on the PCIA pump can greatly mask the synergistic analgesic effect of adjuvants in the early postoperative period. There are several potential mechanisms related to the analgesic effect of DEX. Many studies have shown that DEX mainly exerts analgesic effects through three levels: it acts on the locus coeruleus α_2_AR at the level above the spinal cord, activates the descending noradrenergic inhibitory system, and then enhances the inhibitory synaptic transmission in the dorsal horn of the spinal cord and relieves pain; at the level of the spinal cord, it binds to α_2_AR in the dorsal horn of the spinal cord, causing cell membrane superization, thereby inhibiting the upward conduction of pain signals; at the peripheral level, it inhibits Ad fibers and C fibers, preventing them from transmitting nociceptive information to the spinal cord (Grape et al., 2019; Bozorgi et al., 2021).

Our study found no statistical difference between DEX and analgesia alone in the RSS sedation score, which may be related to the lower dose of DEX for PCIA. Relevant studies have confirmed that surgery, anxiety, pain and other strong stimuli are associated with sympathetic nervous system excitation. DEX can activate the central locus coeruleus receptor, reduce sympathetic tone, and reduce the release of epinephrine and norepinephrine, resulting in a sedative effect (Qiu et al., 2016).

The total amount of analgesics supplemented in the DEX group 24 h after surgery was less than that in the control group, and the times of use of rescue analgesics in the DEX group was also less than that in the control group. This indicates that the addition of DEX to PCIA exerts a better analgesic effect, and has a synergistic effect with analgesic drugs. Analgesics-related complications were significantly reduced, while patient satisfaction was improved in the DEX group, compared with PCIA with analgesics alone. These changes may be explained as follows: 1) patients receiving DEX for PCIA used lower doses of analgesic drug; 2) DEX reduces noradrenergic activity by activating presynaptic α_2_ receptors in the locus coeruleus or reducing sympathetic outflow, which may cause postoperative nausea and vomiting (Choi et al., 2017).

IL-6 is a lymphokine produced by activated T cells and fibroblasts. It can make B cell precursors become antibody-producing cells, cooperate with colony-stimulating factors, can promote the growth and differentiation of original bone marrow-derived cells, and enhance the lysis function of natural killer cells (Tanaka et al., 2014; Tanaka et al., 2018). Th2 cells mainly secrete IL-4 and IL-6. DEX group reduced the release of IL-6, polarize Th2 cells to Th1 cells and relieve the suppression of cellular immunity caused by surgical stress. TNF-α is a substance that can cause hemorrhagic necrosis in various tumors appears in serum. It can kill or inhibit tumor cells, promote cell proliferation and differentiation, which is a key pro-inflammatory cytokine (Robinson et al., 2020). The DEX group reduces the release of TNF-α, thereby exerting an anti-inflammatory effect.

The ability of DEX to reduce plasma levels of TNF-α and IL-6 to reduce the inflammatory response has been demonstrated in several studies (Taniguchi et al., 2004; Qiao et al., 2009; Tasdogan et al., 2009). However, the immunosuppressive effect of DEX on patients is also noteworthy. Su et al. (2018) found that DEX induced proliferation of monocytic myeloid-derived suppressor cells (M-MDSC), a cell population with a strong pro-angiogenic capacity, in lung cancer patients after surgery, and that DEX expanded M-MDSC in mice and promoted tumour metastasis by increasing VEGF production. Smith et al. (2014) similarly focused on the suppressive effects of DEX on the immune system. Interestingly, Lee et al. (2021) used the immunosuppressive effect of DEX to mitigate allograft rejection and prolong graft survival in mice. However, overall, DEX immunosuppression-related articles are scarce, there is currently insufficient attention to its immunosuppressive effects, and more high-quality studies are needed in the future to assess the impact of the immunosuppressive effects of DEX on patient prognosis.

Hypotension and bradycardia were considered major concerns regarding the safety of adding DEX to postoperative intravenous PCA (Carollo et al., 2008), and therefore DEX should be used with caution in patients with severe bradycardia and hypotension. In addition, in patients with stroke or coronary artery disease, the hypotensive or bradycardic effect of DEX is strongly associated with prognosis (Sousa et al., 2021). When used at clinically recommended concentrations, DEX produces dose-dependent hypotension and bradycardia due to inhibition of sympathetic neurotransmission and a decrease in sympathetic tone, this effect may also be caused by baroreceptor reflexes and mediated by enhanced vagal activity (Ebert et al., 2000; Paris and Tonner, 2005; Frolich et al., 2011). In this study, the risk of bradycardia was higher in the DEX group than in the control group, but the risk of hypotension was not statistically different between the two groups, of course, none of the included patients did not require medical correction for the reduced heart rate. However, studies have shown that DEX can inhibit corticotropin-stimulated corticosterone release after prolonged or high-dose use (Maze et al., 1991), but the studies we included did not observe the effect of DEX in PCIA on this indicator, and more clinical studies are necessary to verify in the future. In addition, rebound hypertension and tachycardia after abrupt discontinuation of DEX, changes in tolerability, and the possibility of discontinuation syndrome are also of concern (Feng et al., 2019).

In the past, there were two meta-analyses on the effectiveness and safety of DEX for PCAI. Chen et al. (2020) found that DEX combined with tramadol for PCIA was better than tramadol alone in terms of analgesic effect and safety. Peng et al. (2017) found that DEX was an effective adjuvant to opioid PCIA, which could reduce postoperative pain, opioid demand and opioid related adverse events of patients. Apart from the tramadol and pure opioid agonists (Morphine, Sufentanil, Fentanyl, Oxycodone), our meta-analysis also included studies of butorphanol and ketorolac for PCIA. And our meta-analysis included the largest number of studies (28) of DEX for PCIA to date. In addition, our meta focused on the effect of DEX in PCIA on the postoperative inflammatory response of patients, the area that has not been the focus of previous meta-analyses. Thus, our meta-analysis provides a comprehensive assessment of the efficacy and safety of DEX in PCIA, providing a reference for the clinical application of DEX in PCIA.

There are several limitations in our meta-analysis. First, we may have missed some studies that met the inclusion criteria and had to exclude some studies because the full text was not available. Second, this meta-analysis included several different types of analgesic drugs (morphine, sufentanil, fentanyl, oxycodone, tramadol, etc.) that may affect pain relief outcomes and adverse events. Third, some of our results have large heterogeneity, which may lead to biased results and requires further study. The reasons for the high heterogeneity may be related to different analgesic drugs used in PCIA, different doses of DEX, and different PCIA parameter settings. Finally, for the larger heterogeneity results, although we tried subgroup analysis and sensitivity analysis, we were still unable to reduce their heterogeneity.

## Conclusion

Compared with analgesics or placebo alone, DEX combined with postoperative PCIA can achieve better analgesia and patient satisfaction, while reducing analgesics consumption and the occurrence of analgesics-related adverse events.

## Data Availability

The original contributions presented in the study are included in the article/Supplementary Material, further inquiries can be directed to the corresponding authors.
